# The impact of living conditions and health interventions on tuberculosis, Denmark, 1876 to 2022

**DOI:** 10.2807/1560-7917.ES.2024.29.24.2300652

**Published:** 2024-06-13

**Authors:** Anne Christine Nordholm, Anja Joergensen, Louise Hedevang Holm, Aase Bengaard Andersen, Anders Koch, Peter Henrik Andersen, Troels Lillebaek

**Affiliations:** 1 Department of Infectious Disease Epidemiology and Prevention, Statens Serum Institut, Copenhagen, Denmark; 2 International Reference Laboratory of Mycobacteriology, Statens Serum Institut, Copenhagen, Denmark; 3 Department of Infectious Diseases, Rigshospitalet Copenhagen University Hospital, Copenhagen, Denmark; 4 Department of Public Health, University of Copenhagen, Copenhagen, Denmark; * These authors contributed equally to the work and share first authorship.; ** These authors contributed equally to the work and share last authorship.

**Keywords:** Epidemiology, public health, disease surveillance, morbidity, mortality, tuberculosis, infection control, surveillance, epidemiology, Denmark

## Abstract

**Background:**

Denmark possesses an exceptional historical data collection on tuberculosis (TB) from 1876 to the present, providing a unique opportunity to assess TB epidemiology over 147 years in Denmark.

**Aim:**

Our aim was to describe the TB disease burden in Denmark in relation to historical events, living conditions and health interventions during the past 147 years.

**Methods:**

We performed a nationwide register-based ecological study including all persons with TB in Denmark from 1876 through 2022, correlating the TB incidence to social, economic and health indicators.

**Results:**

In Denmark, the overall TB incidence and mortality declined markedly over the past 147 years, only marginally influenced by specific TB interventions such as sanatoria, Bacillus Calmette-Guèrin (BCG) vaccination, mass screenings and antibiotics. Parallel to this decline, the country experienced improved living conditions, as illustrated by decreased infant mortality and increased life expectancy and wealth. In 1978, Denmark became a low-incidence country for TB with risk groups predominantly affected, and with a continuous change in demographics towards fewer Danish-born cases and relatively more migrant cases.

**Conclusions:**

The decline over time in TB incidence and mortality in Denmark preceded specific TB interventions and can, first of all, be attributed to improved living conditions. TB has now become a rare disease in Denmark, predominantly occurring in particular risk groups. Future elimination of TB will require a combination of specific health interventions in these risk groups combined with a continued focus on improving socioeconomic status and living conditions.

**Key public health message**

**What did you want to address in this study and why?**

Tuberculosis (TB) is a multifactorial disease that is present all over the world but highly unevenly distributed both across countries and within countries. Our aim was to discuss various TB interventions and how these related to the socioeconomic context over time in Denmark.

**What have we learnt from this study?**

The TB burden decreased remarkably in Denmark over the past 150 years, and TB is now a rare disease in the country. Interestingly, this decline over time in TB incidence and mortality preceded specific TB interventions such as BCG vaccination, public screening and antibiotic treatment. These important health interventions did not individually impact the TB burden, and we assume that the decline in TB was mainly due to improved living conditions in Denmark.

**What are the implications of your findings for public health?**

Tuberculosis is a disease of the poor and disadvantaged population, and the future approach against TB must focus equally on health interventions, easy and equal access to care, and on improving health status and living conditions worldwide.

## Introduction

Tuberculosis (TB) has occurred globally for centuries and remains a major public health problem. As in many other European countries, TB used to be endemic in Denmark, but during the past approximately 150 years, there has been a steep decline in the TB incidence. In 1978, Denmark became a low-incidence country for TB with less than 10 cases per 100,000 population per year. Over the years, great medical advances have been made such as the development and distribution of the Bacillus Calmette-Guérin (BCG) vaccine and the introduction of effective anti-tuberculous drugs. In Denmark, these advances have coincided with substantial economic growth and improved living conditions as well as the implementation of major public health interventions including mass TB screenings and the eradication of bovine TB [1]. Tuberculosis is now a rare disease prevailing mainly in specific risk groups in the society including migrants [2] and socially deprived residents [3,4]. However, the mortality among people with TB remains substantial [5].

Different factors have impacted the TB incidence and mortality over the past two centuries. In this article, we explore original TB data dating back to 1876, when it became mandatory to notify TB mortality in Danish towns [6]. In 1921, it became mandatory to notify all patients diagnosed with TB to a national registry [6]. These historical data provide a unique opportunity to describe the epidemiology of TB for an entire nation spanning nearly 150 years. It enables us to discuss the TB disease burden in Denmark over time and relate epidemiological changes to historical events, social conditions and health interventions.

## Methods

### Setting and study design

This is a nationwide retrospective register-based ecological study on the epidemiology of TB in Denmark spanning 147 years from 1876 through 2022, starting with the first officially available TB statistics in the form of local mortality rates. In 1921, the national TB surveillance registry (TBSR) was established when it became mandatory to notify all cases of pulmonary TB (PTB) to the health authorities, and in 1951, the notifications were expanded to include extrapulmonary TB (EPTB). The TBSR has developed from containing aggregated numbers of TB cases to include individual-level information about demographics and disease manifestations.

### Data collection

**Figure 1:**
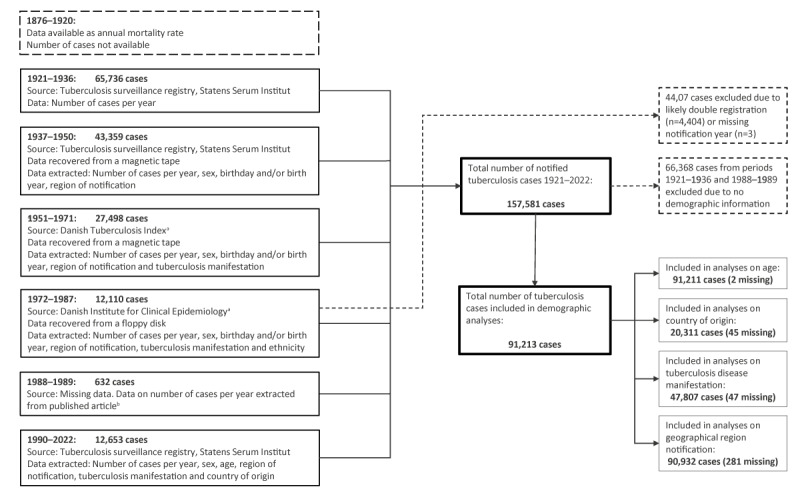
Tuberculosis data collection from different registers, Denmark, 1921–2022 (n = 157,581 cases)

^a^ Danish Tuberculosis Index changed name to Danish Institute for Clinical Epidemiology in 1974. In 1999, it changed to National Institute for Public Health.

^b^ Poulsen, S. et al. Tuberkuloseudviklingen i Danmark 1972–1996. Ugeskr. Laeger, 1999 [7].

Data collection is illustrated in detail in Figure 1. Data from 1876 through 1920 were extracted from published TB mortality rates [6]. From 1921 through 2022, data were retrieved from the TBSR. For the period 1921 to 1936, the TBSR consists of aggregated data with annual number of cases. Since 1937, the TBSR has contained individual data on birth date or year of birth, sex, and geographical region of Denmark. In 1951 and in 1972, information on TB manifestations and country of origin were included, respectively. A graphical representation of mortality rates from 1876 to 1950 is appended in Supplementary Figure S1.

The different registries over time have been gathered in the national tuberculosis surveillance registry (TBSR) centrally hosted at the Department of Infectious Disease Epidemiology and Prevention, Statens Serum Institut.

For the period 1937 to 1950, the TBRS contained slightly fewer cases per year than some of the published statistics [6]. This is most likely explained by data from these years being lost and subsequently recovered in 2003. Our analyses are based on the TBRS, except for the years 1988 and 1989 for which the original data have been lost. We retrieved absolute numbers from these two years from a previously published article on TB in Denmark [7]. Two cases had missing data on age, 47 on TB manifestations, 45 on country of origin and 281 on geographical region of notification.

From Statistics Denmark, we extracted publicly available information on annual population numbers and on indicators of the living standard in Denmark including life expectancy, infant mortality and gross domestic product. Information on TB interventions, policies and legislations were collected from publicly available sources to discuss potential correlations with TB incidence, disease burden and mortality.

### Definitions

We stratified people with TB into three groups by geographical origin: Danes, Greenlanders and migrants. Danes were defined as people born in Denmark with both parents also born in Denmark. Greenlanders were defined as people born in Greenland or people born in Denmark whose parents (one or both) were born in Greenland. Greenland is an integral part of the Kingdom of Denmark with self-rule. Greenlanders automatically obtain Danish citizenship at birth, and ca 17,000 Greenlanders live in Denmark. Until 1987, people of Greenlandic origin were registered as Danes and are therefore categorised as such in our analysis up to 1987. Migrants were defined as people born outside of Denmark and Greenland or people born in Denmark whose parents (one or both) were born outside of Denmark and Greenland.

We stratified geographical areas of notification into the five regions of Denmark: The Capital Region, Region Zealand, and the Northern, Southern and Central Region.

### Statistical analyses

In the 1920s, before antibiotics were available, the TB incidence was approximately twice that of the mortality [6]. Based on the assumption that this also applied to the time period before the 1920s, were calculated the annual TB incidence rates for the years 1876 through 1920 as twice the annual mortality rate. Annual TB incidence was calculated from 1921 through 2022 with the population of Denmark per 1 January as the denominator. We calculated accumulated age cohorts to demonstrate age group incidences over time. For categorial variables, data are presented as numbers and percentages, and the chi-squared test was used for data comparisons. We calculated median age with 25–75% interquartile range (IQR) and used the Wilcoxon rank sum test for comparisons.

In case of missing data, the cases with missing information (such as age or TB disease manifestation) were not included in the stratified analyses but appear in the total numbers of TB cases by year.

Over the study period, data from different sources were initially merged into a database in Excel. All further data management and statistical analyses were subsequently performed using R version 4.3.2.

## Results

**Figure 2:**
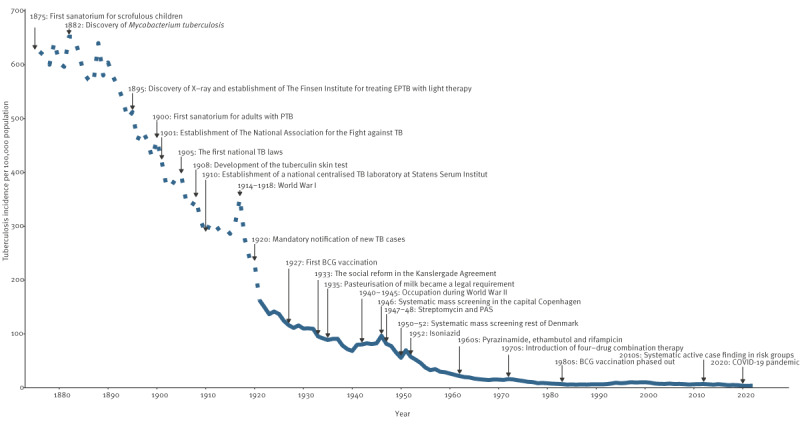
Tuberculosis incidence, Denmark, 1876–2022, and public health interventions and historical events

BCG: Bacillus Calmette-Guérin; EPTB: extrapulmonary tuberculosis; PAS: para-aminosalicylic acid; PTB: pulmonary tuberculosis; TB: tuberculosis; WWII: World War II.

Overall, the TB incidence in Denmark declined remarkably from 1876 through 2022 (Figure 2) with peaks around the two world wars and the systematic mass TB screenings of the Danish population in 1946 and 1951. The highest TB incidence was recorded in 1883 (652/100,000 population) vs the lowest in 2021 (4/100,000 population).

**Figure 3:**
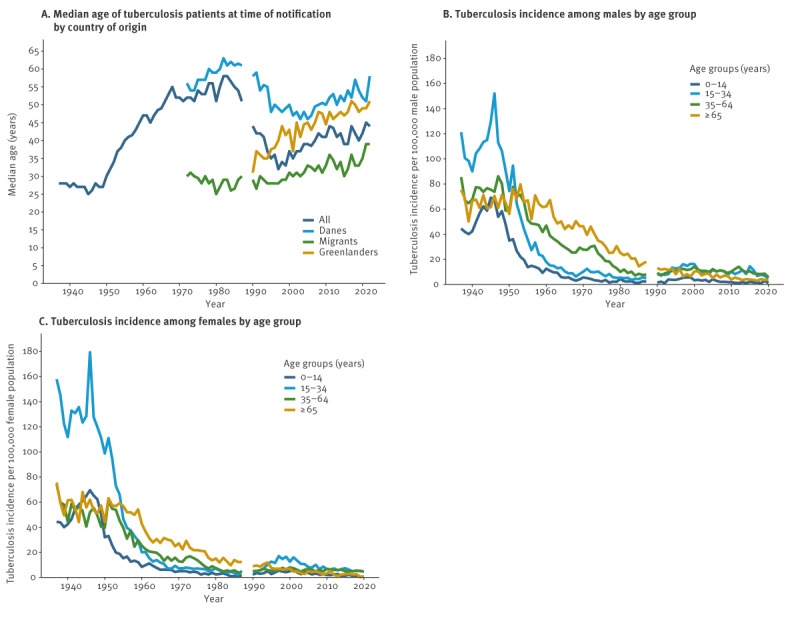
Age curves of persons notified with tuberculosis, Denmark, 1937–2022 (n = 91,211)

**Figure 4:**
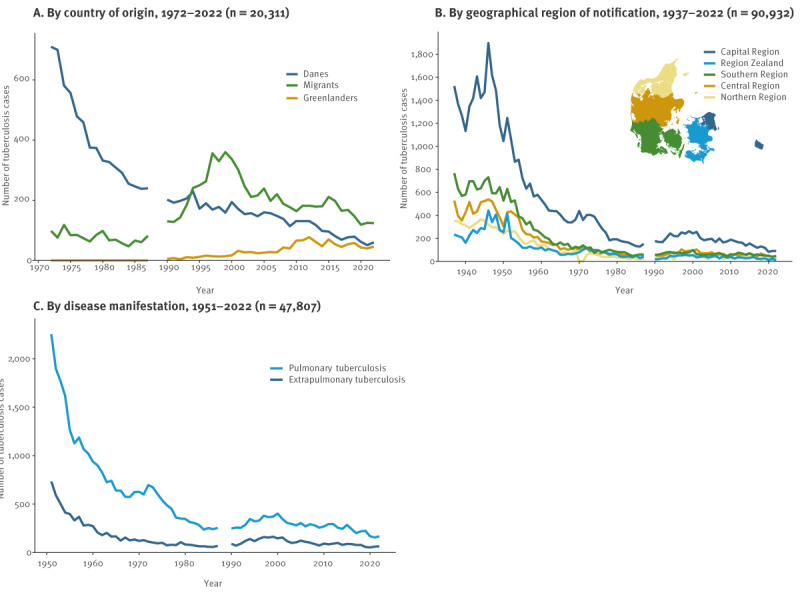
Demographic characteristics and disease manifestations of persons notified with tuberculosis, Denmark, 1937–2022

Additional demographic information was available from 1937 through 2022 for 91,213 cases (Figure 1). The median age for people notified with TB fluctuated over this period (Figure 3A). In the 1930s and 1940s, the median age was around 25 years, then it increased notably and peaked at 58 years (IQR: 32–72) in 1982, after which it decreased and levelled around 40 years. The highest age group incidence was observed among 15–34-year-old men and women in the 1940s and 1950s shifting towards older age groups from the 1960s onwards (Figure 3B-C). Up until the 1990s, TB was predominantly found among Danes, constituting 80% of cases. However, this proportion decreased markedly and since 1994, more migrants than Danes have been notified with TB (Figure 4A). At present, Danes constitute ca 25% of notified TB cases. The observed changes in median age over time was associated with the cases’ country of origin (Figure 3A). The median age of migrants notified with TB was significantly lower than that of Danes (30 vs 55 years; p < 0.0001). 

From 1937 to 1955, approximately the same share of men and women were notified with TB. However, in the late 1950s, this gradually changed towards a noticeable predominance of men, at present around 60%.

The geographical distribution of TB cases in Denmark is illustrated in Figure 4B. During the whole observation period, the vast majority of cases occurred in the capital region compared with other regions in Denmark (45.9% of cases vs 9.5–19.7%; p < 0.0001).

**Figure 5:**
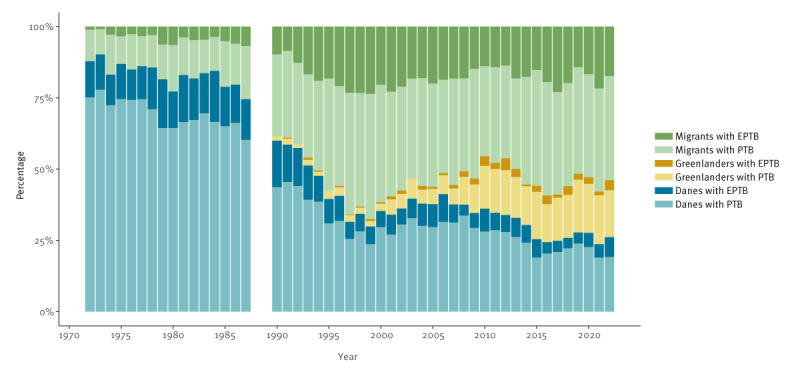
Share of pulmonary and extrapulmonary tuberculosis among people notified with tuberculosis, by country of origin, Denmark,1972–2022 (n = 20,264)

EPTB: extrapulmonary tuberculosis; PTB: pulmonary tuberculosis; TB: tuberculosis.

The majority of TB cases were PTB (Figure 4C), with a median proportion of EPTB of 22.6% over the time period. Migrants had a significantly higher rate of EPTB than Danes and Greenlanders (32.0% vs 17.5% and 14.1%; p < 0.0001) (Figure 5).

**Figure 6:**
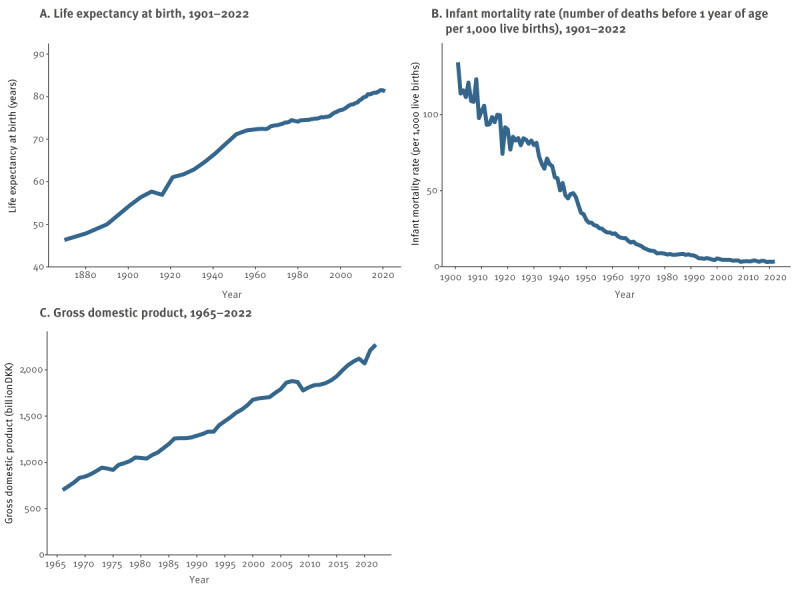
Indicators of living standard, Denmark, 1876–2022

Throughout the study period, the living standard in Denmark increased remarkably, as illustrated in Figure 6. There was continuous economic growth with an increase in the national gross domestic product. Life expectancy increased from 48 years in 1880 to 81 years in 2022, and infant mortality concurrently decreased from 134 to three per 1,000 live births between 1901 and 2022.

DKK: Danish krone.

## Discussion

In this nationwide register-based study spanning nearly 150 years, Denmark changed from a TB high-incidence to a TB low-incidence country, coinciding with considerable demographic changes among people notified with TB. Interestingly, important health interventions did not individually impact the TB disease burden over time in Denmark as TB incidence, and the decrease in mortality was mostly due to improved wealth, living conditions and better health status of the Danish population.

Especially during the first 100 years, a steep decline in TB incidence and a shift towards fewer cases among children were observed. During the last ca 50 years, the old TB epidemic transformed into a disease of risk groups. Today, TB occurs predominately among migrants and socially disadvantaged persons.

Initially, TB treatment focused on isolation of patients in sanatoria. In 1875, the first treatment institution opened for children with scrofulous disease and in 1900, the first sanatorium for PTB patients opened [6]. Several sanatoria were established in the following years, and treatment consisted of healthy meals, moderate walking exercise and rest in open air [6]. People with TB were separated from their families, some lost their jobs and income, and children missed school education, resulting in TB-related stigma. After Niels Finsen’s discovery of light therapy in 1892, this was added to the treatment of EPTB, using carbon arc light and mercury lamps [6].

In 1901, the first national TB control programme was implemented in Denmark, after some prominent physicians and politicians established *The National Association for the Fight against Tuberculosis* [6]. In 1905, the first TB laws were introduced, including public financing of TB management and the establishment of TB dispensaries for surveying and treating TB locally [8]. Patients suspected of TB were referred to the TB dispensaries. After the invention of X-ray, the tuberculin skin test (TST) and the BCG vaccine, the national TB management was extended to include a preventive approach with screening and vaccination [6]. The TB dispensaries performed X-rays, blood samples and contact investigations [6]. In 1927, the first person in Denmark was vaccinated with BCG: a child born to a mother with PTB. However, it was not until 1945 that BCG vaccination was systematically introduced in schools and became part of the national childhood vaccination programme. In 1941, Denmark acquired its first X-ray bus [8] used to promote active case finding, and in 1946, the first TB mass screening campaign took place in the capital, Copenhagen, with 60% of the population being screened, causing a peak in the TB incidence [9]. In the years 1950 to 1952, a similar screening campaign was implemented in the rest of the country, also evident in the TB statistics. Following the success of these TB mass screenings, the dispensaries continued annual screenings of nearly 1 million people throughout the 1950s and 1960s [10] with more than 90% of TST-negative schoolchildren being vaccinated in the first 3 years [1]. Interestingly, we found a 46% reduction in notified TB cases among children aged 5–14 years in the 1950s compared with the 1940s, while the reduction among children aged 0–4 years (who were most likely unvaccinated) was only 16.6%, indicating an effect of the vaccination programme on childhood TB. Overall, there was a remarkable shift in age among people with TB towards older age groups around the 1950s in Denmark. This mirrors observations from Finland and Sweden [11], and most likely represents lower transmission rates and a higher proportion of cases resulting from reactivation of TB infection acquired earlier in life.

When antibiotics with anti-tuberculous effect were introduced in the 1940s, TB gradually became a curable disease. The introduction of combination therapy in the 1970s coincided with a continued reduction in the TB mortality [10]. In other European countries and in the United States, the mortality rate declined before the introduction of TB-specific antibiotics, whereas for Brazil, Japan and South Africa, the decline was observed later and mainly related to antibiotic treatment of TB cases [12,13]. Undeniably, antibiotic treatment had a major impact on TB mortality, but the decrease in TB incidence was primarily influenced by other factors. 

Another major health intervention was the elimination of bovine TB, following its recognition in the 1880s as source of human infection. An eradication effort was made using TST, isolation of infected animals and pasteurisation of milk [6,8]. In 1932, these efforts were intensified, leading to an immediate and remarkable decrease in bovine TB. In 1952, bovine TB was officially eradicated in Denmark [1].

As a consequence of the declining TB incidence, the yearly mass screenings were replaced by passive case finding and contact tracing in the 1970s [10]. This coincided with an increasing number of sputum smear-positive individuals from 28% in 1972 to 47% in 1978 [10], indicating diagnostic delay. Still, the overall TB incidence continued to drop even despite the concurrent HIV/AIDS epidemic that caused a global surge in TB incidence but had little impact on TB in Denmark. In the 1980s, the last TB dispensaries closed and BCG vaccination was phased out of the national vaccination programme. Management of TB patients was centralised in hospitals at infectious disease and pulmonology departments or, for children, at paediatric departments [14]. 

It is remarkable that the decline in TB incidence preceded the introduction of specific health interventions, the sanatoria treatment, BCG vaccination, TB screening and antibiotic treatment. The rapid decline in TB incidence in the late 1800s seems to have a striking synchronicity with significant improvement in living conditions and general health status in Denmark. This is consistent with findings from other European countries and the United States [12]. In the late 1800s and early 1900s, a large proportion of the population lived under poor housing conditions with crowding and poor ventilation, providing optimal conditions for TB to spread and flourish. At the turn of the century, there were on average five inhabitants per household with ca 12 m^2^. per person [15]. By 2020, 120 years later, there were two inhabitants per household and 52.8 m^2^. per person [16]. Concurrently, hygiene and sanitary conditions improved markedly. Clean water supplies and urban sewer systems were introduced in the mid-1800s to combat cholera epidemics, with a major impact on people’s health in general. The nutritional status improved, and the average height increased in the Danish population, reflecting a better state of health [17]. In 1933, the landmark social reform, *The Kanslergade Agreement*, was introduced aiming to provide social welfare benefits to those in need. The reform is considered the cornerstone in the foundation of the Danish welfare state. Another indication of the importance of the role of nutrition and general living conditions on TB incidence is the impact of the two world wars. The TB incidence and mortality rose markedly, most likely due to shortages in supplies during the war years [8].

Although no data on the origin of TB patients exist until 1972, migration did influence TB in Denmark before that date, e.g. around 350,000 German civilians fled to Denmark after the Second World War, living in camps affected by communicable diseases including TB until 1949. Furthermore, it has been suggested that the introduction of the TB strain ‘Cluster 2’ to Denmark happened with a group of Hungarian refugees in 1956 [18]. Cluster 2 has since led to the largest newer outbreak of TB in the country since 1992, with more than 1,000 cases. Migration accelerated with the economic boom in the 1960s [19], but it was not until the 1990s that it had a major impact on TB incidence. The observed increase in TB cases in Denmark in the 1990s was particularly driven by migrants from Africa, especially Somalia. Of the 13,535 Somali people migrating to Denmark in the 1990s, 6.7% developed TB and accounted for 21.7% of all TB cases during that time period [20]. A study of TB among migrants from 1993 through 2015 found that migrants overall had a 30 times higher risk of developing TB than Danes [2]. Genomic typing has shown that most migrants are infected before entering Denmark [21] and only limited transmission occurs between migrants and Danes [7,21].

It is worrying that while they represent only 0.3% of the population in Denmark, Greenlanders constitute around 20% of TB cases [22]. The reason for this is likely multifactorial. Contrary to Denmark, Greenland is a setting with high TB incidence and in addition, the integration into Danish society can be difficult. Because they do not have migrant status, Greenlanders do not receive the same help as other migrants, and a large group of Greenlanders are socially marginalised [23].

A concern about migration to Denmark from countries with high TB incidence is the risk of multidrug-resistant (MDR) TB, resistant to isoniazid and rifampicin. In some countries, MDR-TB accounts for 20% of all new TB cases [24]. Especially eastern European and central Asian countries have high burdens of MDR-TB. Since the expansion of the European Union in 2004, large groups of migrants have come to Denmark from eastern Europe [19]. Up until 2022, MDR-TB was not a major problem in Denmark [25] constituting less than 2% of TB cases but in 2022, an exceptionally high number of 12 MDR cases was observed corresponding to 5% of TB cases [26]. All except one case were migrants from countries with a high burden of MDR-TB. Preliminary numbers for 2023 showed continuously high numbers of MDR-TB cases (data not shown), suggesting a permanent rise in resistance that needs to be addressed in future TB management.

Despite the current low TB incidence in Denmark, there are notable disparities, as most cases occur in risk groups including migrants from countries with a high TB burden and socially deprived persons [2,3,27] and there is ongoing transmission especially among middle-aged Danish and Greenlandic marginalised men who are homeless or have a substance use disorder [22,28]. The Danish healthcare system offers free, equal and universal access for all residents. Still, social and health inequity exist with TB as a very sad example of a social disease that continues to prevail among the most vulnerable and marginalised populations of the society.

This study was based on nationwide surveillance data uniquely dating back 147 years. Strengths include long study period, comprehensive surveillance data and nationwide design. However, there are some limitations. The estimated incidence during the period 1876 through 1920 is subject to some uncertainty. The incidence was calculated as twice the mortality rate from the early 1920s when the TB incidence was approximately double the mortality [6]. However, as shown above, the living conditions and general health improved markedly during the early part of the study period. Thus, the mortality among people with TB could potentially have been even higher than 50% in the late 1800s resulting in an overestimation of the TB incidence. However, our results are consistent with the locally described trends in TB incidence and give a fair estimate of the TB incidence nearly 150 years ago.

## Conclusions

Over the past nearly 150 years, TB incidence and mortality have decreased remarkably in Denmark. Simultaneously, there have been important economic growth and improved living conditions as well as major public health interventions targeting TB, such as TB screenings, BCG vaccinations and specific TB treatment. The decline in TB incidence preceded the introduction of TB-specific interventions, illustrating the importance of, in particular, improved living conditions for reducing TB risk. Even in the 21st century, TB is still a disease of the poor and disadvantaged. Future action against TB must contain equal focus on socioeconomic and health interventions.

## Supplementary Data

305000 Supplement
