# Supplementary material for: The impact of living conditions and health interventions on tuberculosis, Denmark, 1876 to 2022
Source: Euro Surveill. 2024 Jun 13;29(24):2300652. doi: 10.2807/1560-7917.ES.2024.29.24.2300652 (PMC11177568; doi:10.2807/1560-7917.ES.2024.29.24.2300652)
Supplement: Supplement [file 23-00652_NORDHOLM_Supplement.pdf]

This supplementary material is hosted by Eurosurveillance as supporting information alongside the article *The impact of living conditions and health interventions on tuberculosis, Denmark, 1876 to 2022*, on behalf of the authors, who remain responsible for the accuracy and appropriateness of the content. The same standards for ethics, copyright, attributions and permissions as for the article apply. Supplements are not edited by Eurosurveillance and the journal is not responsible for the maintenance of any links or email addresses provided therein.

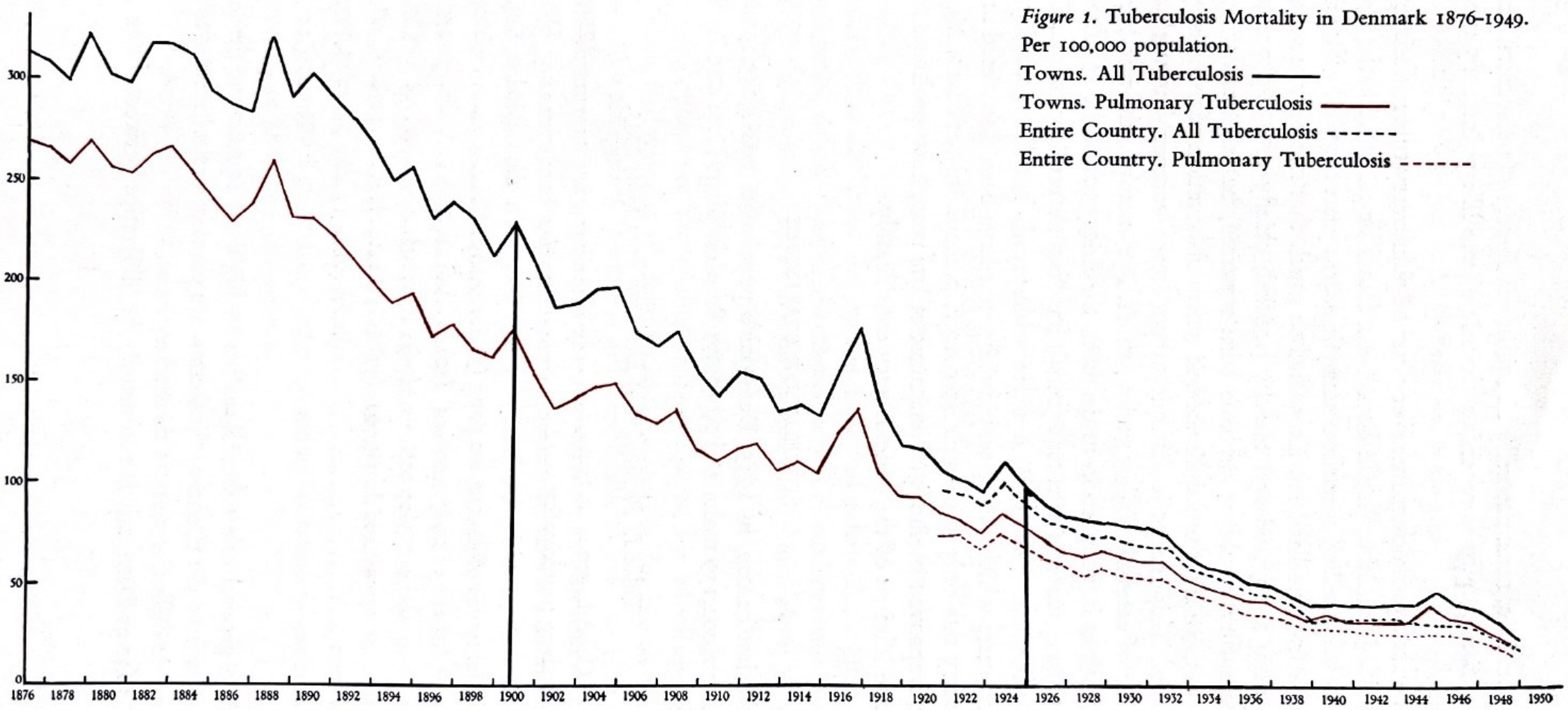

Figure reprinted from The National Association for the Fight against Tuberculosis. The fight against tuberculosis in Denmark. Copenhagen: Nyt Nordisk Forlag Arnold Busck; 1950 with permission from Lungeforeningen (The Danish Lung Association – formerly The National Association for the Fight against Tuberculosis)

The yearly mortality rate was read from this figure, and the incidence of tuberculosis for the period 1876 through 1920 was subsequently calculated as the mortality rate per year times 2.
